# From knowing our needs to enacting change: findings from community consultations with indigenous communities in Bangladesh

**DOI:** 10.1186/s12939-015-0264-x

**Published:** 2015-11-09

**Authors:** Sameera Hussain, Ana Lorena Ruano, Atiya Rahman, Sabina Faiz Rashid, Peter S. Hill

**Affiliations:** James P Grant School of Public Health, BRAC University, Dhaka, Bangladesh; School of Population Health, University of Queensland, Brisbane, Australia; Department of Global Public Health and Primary Care, Center for International Health, University of Bergen, Bergen, Norway; Centro de Estudios para la Equidad y Gobernanza en los Sistemas de Salud, Guatemala, Guatemala; BRAC, Dhaka, Bangladesh

**Keywords:** Bangladesh, Indigenous communities, Go4Health, Marginalization, Determinants of health, Chittagong Hill Tracts

## Abstract

**Introduction:**

Indigenous peoples are among the most marginalized peoples in the world due to issues relating to well-being, political representation, and economic production. The research consortium Goals and Governance for Global Health (Go4Health) conducted a community consultation process among marginalized groups across the global South aimed at including their voices in the global discourse around health in the post-2015 development agenda. This paper presents findings from the consultations carried out among indigenous communities in Bangladesh.

**Methods:**

For this qualitative study, our research team consulted the Tripura and Mro communities in Bandarban district living in the isolated Chittagong Hill Tracts region. Community members, leaders, and key informants working in health service delivery were interviewed. Data was analyzed using thematic analysis.

**Findings:**

Our findings show that remoteness shapes the daily lives of the communities, and their lack of access to natural resources and basic services prevents them from following health promotion messages. The communities feel that their needs are impossible to secure in a politically indifferent and sometimes hostile environment.

**Conclusion:**

Communities are keen to participate and work with duty bearers in creating the conditions that will lead to their improved quality of life. Clear policies that recognize the status of indigenous peoples are necessary in the Bangladeshi context to allow for the development of services and infrastructure.

## Introduction

Indigenous peoples are among the world’s most marginalized population in terms of social issues, political representation, and economic production [1–3]. Often isolated from non-indigenous culture, they hold separate histories, languages, and traditions. There are 370 million indigenous people in the world, with 70 % of them concentrated in Asia. Notwithstanding this, there are few policies in place that have successfully improved these groups’ access to healthcare and to other social services [4]. In addition, their lack of representation at every level of government ensures that their voices remain unheard and that their needs are not seen as a priority. Despite the existence of global policies and covenants that directly deal with indigenous people’s rights, few countries in the region have recognized their status and their need for special policies aimed to breach the economic, social and health-related gaps between indigenous and non-indigenous populations [5].

Bangladesh is one of the countries in the Asian region that that does not recognize the status of indigenous peoples, instead referring to the many ethnic groups living on ancestral lands as ‘tribal people’. The country commonly refers to these groups by their geographical location, or ‘pahari’, which means hill people, or by their occupation, ‘jumma’, a word that designates a person involved in the local agricultural practice of shifting cultivation, called ‘jum’ [6–9]. Although the country’s 1997 peace treaty specifically calls for the self-determination and promotion of the economic, social, cultural, and civil rights of ‘pahari’ people, Bangladesh continues to exclude them from civil and political life [9]. Despite this marginalization, health indicators in Bangladesh have improved considerably over the last 15 years, which has made it stand out as a paradox in development-related discourse [4]. Bangladesh has been able to meet many targets related to the Millennium Development Goals (MDGs). However, indigenous people remain isolated and marginalized, and their health conditions have not improved in the same way as those of the general population [10].

Aside from a few reports and studies commissioned by the international donors [11–14], little is known about the health and the social determinants of the health of indigenous groups of Bangladesh. Literature around such groups is often limited to official and sanitized accounts, with conflicting reports emerging only sporadically from lawyers and other groups protesting violations of the 1997 Accord [15]. It has been documented that there is some public health outreach in the Chittagong Hill Tracts (CHT) where the majority of Bangladesh’s indigenous communities have traditionally lived [12]. However, services do not reach the remote regions of the country where most indigenous peoples reside, and when they do arrive, they rarely address specific community needs. This sub-study, conducted by a research team located at James P Grant School of Public Health, BRAC University, is part of a multi-country study looking at the health needs of marginalized populations framed within the Goals and Governance for Global Health research consortium (Go4Health), which aims to present evidence and recommendations for the new health-related, post-2015 development agenda. Our aim was to explore the experience that rural, indigenous Bangladeshis have when trying to access care, which includes their relationships to the health system.

## Methods

### Setting

Bangladesh is a lower middle-income country with a total land area of 130,170 km^2^, and a population of 159 million [16, 17]. A small minority, indigenous groups account for 1.1 % of the population, with most of them are settled in the Chitagong Hill Tracts [18]. Located in the extensive hills of the southeast region, the CHT cover 10 % of national land, or 13,294 km^2^, and consists of three districts: Bandarban, Khagrachuri, and Rangamati. This is an area with steep, rugged mountainous terrain and dense jungles with limited arable land and downstream sources of water including waterfalls and springs. The CHT region was ceded to the British colonial rulers of Bangladesh, then Bengal, and officially annexed the CHT in 1900 [9, 19–21]. Today, its status as an excluded area remains and acts as the basis of the legal and administrative system of the region despite significant political changes in the country.

The CHT are home to at around 11 indigenous groups, each with unique ethnic, linguistic, and cultural attributes. All three of the districts are post-conflict areas that were ravaged by over 25 years of civil unrest, which ended with the 1997 CHT Accord, sometimes also known as the Peace Accord. In it, the CHT are further recognized as a separate area that has its own acknowledged cultural and land rights that specifically target the indigenous groups. Locally, traditional governance structures are recognized and the area has been given greater autonomy, and a regional council [9, 22]. However, the Peace Accord has been largely unimplemented and indigenous communities continue to be subject to ongoing militarization and human rights abuses [6, 18, 21–24].

Out of all three districts, Bandarban is the most remote and least populated district, not only in the CHT but also in the country. Of the total population of 400,000 inhabitants, over half belong to one of the local 8 ethnic groups: Marma, Tanchangya, Chakma, Tripura, Khyang, Bawm, Rakhaine, and Mro [25, 26]. The remaining population is made up of Bengali settlers, who arrived as part of transmigration programs in the 1960s and 1970s that promised land grants, cash, and rations endorsed by the government to encourage nationalization with the purpose of doing away with the diverse ethnic identities of the district [27]. Studies have shown that the Bengali people of Bandarban have regular access to public services and utilities, and routinely have better outcomes in terms of social, economic, educational, and health indicators when compared to non-Bengalis. In regards to government organization in Bandarban, this district has a semi-autonomous government that co-exists with local traditional systems. The rest of the country has a centralized national system of governance [28]. There are 7 *upazilas* in Bandarban, which act as units of government at the sub-district level, and these are further divided into 30 *unions*, the smallest administrative units. In addition to this government structure, there are 98 *mouzas* or revenue units and a total of 1554 *paras* or villages [23].

The health system in Bandarban is the same as that of the rest of Bangladesh in that it is highly pluralistic [29]. Services are delivered by both public and private sector through facility based services, community-based services, and traditional medicine. Missionary organizations, and non-government organizations (NGOs) provide services to the communities, and druggists, homeopaths, traditional healers, and traditional birth attendants (TBAs) are also available in town and sometimes within the communities. Public health service outreach is organized by the Ministry of Health and Family Welfare (MoHFW), which follows the nationalized standard for service delivery, which is often of poor quality and lacks accountability. Accordingly, the district hospital is located in Bandarban’s single urban centre. There are also 7 sub-district health complexes and 27 area clinics that provide public health services [12]. However, these facilities are routinely short of resources and healthcare providers are often absent [30]. The MoHFW has had limited reach in service provision to the communities via satellite clinics or doorstep services, attributing this to the absence of roads [14].

### Study population

Two indigenous communities were consulted for this study, the Tripura and the Mro. Each community resides in paras made up of clusters of households that are ethnically, linguistically, and culturally homogenous. Under the leadership of village headmen and karbaris, traditional leaders whose jurisdictions cover several paras, communities follow their own traditional social hierarchies. Inhabitants of this district also have limited participation in nationalized administrative processes. Both communities have their unique languages. While a few speak Bengali, the official language of Bangladesh, some that carry out commerce in the area may also speak Marma, which acts as a local lingua franca and is the language of the ethnic majority in Bandarban.

The first community selected for this study, Haatibhanga, belongs to the Tripura ethnolinguistic group and is located about a 2 h walk from the nearest paved road. All homes and the *para* center, which doubles as a schoolhouse, are built out of straw and bamboo. There is no electricity, piped water, or sanitation, and residents rely on natural sources of water for drinking and washing. The community is largely self-sufficient: they are subsistence farmers that weave their own clothes, and that have small-scale businesses. The primary source of food and livelihood is shifting cultivation of communal lands.

The second community belongs to the indigenous Mro community, who live in Tonkabati, also referred to as Brickfield. Forcibly relocated to this area in 2006 due to militarization, the community has difficulty accessing arable land and a natural source of water, which has only increased their levels of isolation and marginalization [6]. The new settlement in Brickfield has a para centre, again doubling as a schoolhouse, a small teashop, and a church in the area. Homes are made of straw and bamboo and are built on stilts.

Neither village has a public health facility and instead receive intermittent doorstep services from a Health Assistant or Community Health Care Provider. Two private NGOs also provide doorstep services in the communities through volunteer Community Health Workers (CHWs), and both had a traditional healer and a midwife living among them.

### Data collection

The first step in data collection was to carry out a desk review of secondary data in the topics of health equity, MDGs progress in Bangladesh, and the health situation of indigenous groups living in the Chittagong Hill Tracts in general, and Bandarban in particular. Topic guides for interviews and focus group discussions were structured around Go4Health’s framework of five domains of inquiry, but adapted to the contextual setting. These were: community understandings of health, essential health needs and their provision, determinants of health, the roles and responsibilities of relevant actors, and community participation in decision-making processes [31].

Data collection was carried out by SH and AR during the months of January and February of 2013 in the two previously mentioned villages in Bandarban – Haatibhanga and Tonkaboti. In each one, a team of research assistants with social science backgrounds provided support and helped with interpretation and translation. We used purposive sampling [32], and respondents for community consultations were selected using the expertise of local partner organizations that included a community-based organization in the area, a nationally based organization, and local leaders. Our aim was to ensure the participation of different groups within the community, and not just of local elites. This helped to gather a larger diversity of responses and information about local needs across socio-economic, age and gender divisions.

In addition to gathering community voices, we also carried out interviews with informal health providers, community health workers, community leaders, local government representatives and representatives of community-based organizations. Focus group discussions with respondents from the lay community were organized by sex and age range. In total we conducted 37 interviews. A breakdown of these can be found on Table 1. All interviews and discussions with community members were conducted in the respondents’ preferred language with the assistance of locally recruited interpreters.

**Table 1:** Data collection method and sample by respondent category

Respondent category	Data collection method
Community	FGD [8]
	IDI [8]
Community leaders	IDI [5]
Health providers	IDI [14]
Service delivery organizations	KII [2]

*FGD* Focus Group Discussion; *IDI* In Depth Interview; *KII* Key Informant Interview. The category of Community Leaders includes both traditional and administrative leaders. The Health Providers category includes all people consulted for health care in the community including traditional healers, midwives, homeopaths, community health workers, and druggists. The participation of physicians at the health facilities was sought but they did not wish to participate

### Data analysis

All interviews were recorded with the consent of respondents, and after the fieldwork, they were transcribed in Bengali and then translated into English. We used thematic analysis, a qualitative method used to identify recurring patterns that can be grouped into categories or themes [33, 34]. The first step was to carefully read the transcripts, which was done by AR and SH with feedback and critical inputs from SFR. The second step was to search for the a priori codes from Go4Health’s five domains of inquiry. The nature of qualitative research is that, through it, emerging issues arise from the data. Because of this, emerging codes and categories were also used [35]. During this process, SH and ALR revised and compared codes. Later, the emerging codes and categories were put together with the a priori ones, and emerging themes were obtained from them. ALR, SFR, SH and PH reviewed and agreed on the structure and content of the themes. Finally, themes were narrated and quotes that helped to illustrate the findings were selected.

### Ethical considerations

The ethical review committee of James P Grant School of Public Health, BRAC University, approved this study. In addition to the formal ethical clearance, the researchers also reflected on the social situation that the study participants find themselves in as indigenous people. Excluded from mainstream society, the marginalized communities of Bandarban are denied rights, dignity, and respect, and as a consequence, significant tensions underlie relations with the dominant Bengali majority of Bangladesh. As part of this dominant ethnolinguistic group, the research team recognized its position of power and privilege and understood that as ‘outsiders’ our presence in the communities could be met with apprehension. To mitigate such concerns, the research team used existing partnerships with local NGOs engaged with marginal and socially excluded groups to gain access and entry into the selected communities. These NGOs were staffed by community members with in-depth local knowledge and were trusted by community members and their leaders. These individuals were instrumental in allowing our team to engage with respondents in a meaningful way.

Written consent to participate in the study was not requested from study participants as many did not possess the literacy skills to do so. Instead, consent was sought verbally following the provision of detailed information about the study. To ensure that all participants were fully informed, the research team described the nature, duration, purpose, and methods involved in the research. Only those individuals who gave their voluntary and autonomous consent to participate were interviewed.

## Findings

### Theme 1: Remoteness - life is hard

Describing their local context, participants discussed everyday life as being shaped by the geography of the region. The terrain of steep hills, ridges, and valleys with scarce water and climate conditions affects every aspect of livelihood, lifestyle, and daily activities, and impacts the health of the communities. Participants are aware that other regions in Bangladesh enjoy better living conditions and talked about the absence of basic road infrastructure and public utilities like water, sanitation, and electricity in their villages. They attribute the lack of services to the extreme remoteness of their settlements. The community also discussed the precarious situation they face, particularly in regards to having access to water.

*This is a hilly area; we do not have a proper source of water. Water is scarce. Even if there is some, it is not safe to use water from the waterfall. It is contaminated, especially during the rainy season. As a result, we get diarrhoea and other water-borne diseases.*

For the indigenous people of Bandarban, health is closely related with hygiene and sanitation, two things that are highly valued in the communities. Despite reporting knowledge on these topics, participants said that it is very difficult to put them into practice. This is because water is very scarce and other resources that could be useful in improving the health status of the para are far away from homes. This means that although they are highly valued, washing, bathing, and cleaning are of secondary importance in the remote hills of the region, where there is hardly enough water to drink. As with hygiene, sanitation has also taken a back seat to more pressing needs. During fieldwork, SH and AR observed supplies to build sanitary latrines. However, these remained packed and unused, due to the severe resource constraints that render the latrines unusable.

Social unity is seen as essential for the well-being of the community, a value that is reflected in everyday life, and not only in times of crisis. This applies particularly to the paras’ livelihood. The practice of *jum* farming is central to community life and all adult men and some women walk to the farmlands allocated to them by their local headman. Communal lands are worked using traditional methods. During our discussions, the Mro community reported a drastic change in the distribution of lands in the last decade. This happened as a result of policies implemented by the Bangladesh armed forces. Their forced displacement means that access to natural sources of water and arable land has become limited, adding to the already difficult task of producing enough food, another problem for the indigenous people of Bandarban.

*We were told to leave. We are facing difficulties [here]…we cannot find enough bamboo or trees to cut [for our livelihood] ; the army comes and cuts all the bamboo. We do not have enough food to eat.*

Participants spoke about the difficulty of having a balanced diet that provides them with enough nutrients. The loss of land and their livelihood creates an environment of insecurity. In addition, there is a direct effect on their daily eating habits. It is not only about growing or being able to buy the right food, as storing supplies in the sub-tropical monsoon climate presents complex challenges for the villagers. The paras do not have electricity, and community members spoke about having to cook before every meal in earthen stoves that require firewood in order to avoid gastric problems as a result of eating spoiled food.

*We need to have healthy food that is safe to eat. We cannot eat rotten food. We need to eat food cooked on the same day, or else we suffer from gastric problems. Any food that was cooked earlier – it has to be thrown away.*

As there are no physicians or clinics within reasonable walking distance, there are few options for medical care are available in the community. It is common for villagers to use home remedies or seek the care of traditional healers and TBAs. The community mentioned that they do receive some doorstep services, such as ante- and post-natal care, and health promotion that was provided by the MoHFW and NGOs. However, the services are not regularly rendered and the communities cannot rely on care being readily available. Patients who are seriously ill are taken to town, where they can seek care at either private clinics or at the district hospital, but this is expensive and can create a cycle of debt. These facilities are located 4.5 h walking distance away. Delays in seeking medical care often result in patients growing sicker or even in patient death. Participants reported that it is a 2 h walk to the nearest paved road, the first place where vehicles can finally be used. When patients are extremely sick or weak, the walk to the road is impossible and so community members help to transport the sick using homemade bamboo cages.

### Theme 2: Knowing our needs

During our discussions and interviews, participants expressed their understanding of health as a holistic concept that included basic sanitation, access to food and water and to a social environment that allowed for personal growth. The essential health needs identified can be found in Table 2, which also shows how many times each was mentioned during the course of the fieldwork. When asked about their priorities, the study participants stated that they know what they need for improved health: water and sanitation are of utmost importance, and road access could make these public utilities and other services a reality. However, knowing and prioritizing needs is not enough to improve local situations. Indigenous people still suffer through long days that involve hard agricultural work and long hikes to water sources.

**Table 2:** Ranked essential health needs by respondent categories (*n* = 80)

Essential Health Need	Community people (*n* = 60)	Health providers (*n* = 15)	Community leaders (*n* = 5)
Safe drinking water	60	12	4
Sanitary latrine	51	11	4
Community health centre	45	12	3
Physician	43	8	3
Medicine	32	5	-
Health literacy/behaviour change	31	4	1
Community-based health care worker	20	2	-
Roads and transportation	19	5	1
Clean/hygienic environment	17	2	-
Nutritious food	17	1	-
Income opportunities	10	1	1
Educational institutions	8	2	-
Mosquito nets	7	-	-
Training for traditional birth attendants	1	-	-
Proper housing	1	-	-
Community centre	1	-	-
Electricity	-	-	1

There were few government-provided health services before the signing of the 1997 CHT Accord, and the people in Bandarban did not have access to any public health facility before that year. In the almost 20 years since then, both the Mro and the Tripura that participated in this study recounted the health promotion messages that they have received both from the government and from grassroots organizations and NGOs. These institutions employ health workers that share information about how diseases are spread and why community members must make use of health goods and services. Participants spoke of the health information that is provided by people outside the community, and how this has helped to develop a more biomedical understanding of health. The contradictions and overlapping explanations from both the traditional perspective and the biomedical approach have contributed to creating an environment of tension. Communities recognize the contradictions of being given health promotion messages, despite their lack of access to basic water and government-funded healthcare services.

*They come and advise us to build sanitary latrines, wash our hands before eating food, wear shoes, and wear clothes that are washed and then dried in the sun. If these are maintained, they said, people won’t be afflicted with disease. They suggested these.*

Health promotion campaigns have brought relevant knowledge to the communities, but without enough resources, following recommendations is practically impossible. An example of this is the paras’ drinking water: although the villagers know the importance of boiling water, and know the consequences of not doing so, they lack the time or resources to carry this out at home. As a way forward, participants suggested tube wells or water tanks equipped with lines for communal taps and installed around the village to alleviate the water shortage they regularly experience. Going to gather water from the stream requires significant effort and time investment. Without practical solutions, health advice ticks a box for interventions and programs, but in reality will continue to go unheeded.

### Theme 3: How to enact change

Being part of decision-making processes is important for both communities consulted in this study, and participants expressed that it is only through their active involvement that they can push their collective agenda forward. Meetings are regarded as vehicles for change, as it is in these spaces that local issues are discussed. This is also where decisions in regards to priorities and resource allocation are made. Participants of these meetings include formal and informal community leaders, teachers and health workers. Although this last category enjoys high regard in the paras, thanks to their knowledge and their help in informing communities about pressing concerns, the information they give out is mostly about hygiene and health promotion.

Participants expressed knowing the importance of being part of decision-making processes, and stated that this is a way to enact change within their paras and districts. Although there were mentions of existing mechanisms for participation for non-tribal citizens, there is little movement after communities to use these participation spaces and there are few efforts to include local voices in any decision-making. The villagers felt that the Bangladeshi government shows little interest in the situation, and routinely ignores them:

*They do not try to find out what is happening here. They do not perform their duties. They told us earlier that they would be there for us. But they do not concern themselves with us.*

This population continues to be politically and socially neglected, and government health workers and key decision makers, who are of Bengali ethnicity, are rarely held accountable. Because of this feeling of being ignored, the villagers in both communities consulted for this study feel more comfortable going through NGOs for voicing their needs and concerns. Meetings where villagers are invited to participate are routinely initiated by NGOs, and not the local government. While NGOs open up and provide these spaces, little information is disseminated around official policy processes, and not everyone has a voice that is counted. An elderly woman described the meetings and noted that only some opinions are considered:

*Men participate [at meetings]. If women are invited, they participate. If the meeting is arranged by outsiders [NGOs], women are invited. Everyone’s opinion is heard* [then]*. But in the end they only consider the opinions of those who they think are worthy.*

Despite this, community members see NGOs as the only institutions that are capable of delivering tangible results, and they reported overall improvements in health and education. Traditional leaders also pointed out the importance of NGOs for their communities’ health and wellbeing because they bring with them funding and, although they cannot always deliver the services promised, they are more reliable than the government. However, some participants are cautious about relying solely on them for care. Many of the health and development interventions are project-based, and because they are financed through donors, their presence in the area is only temporal. For the villagers, relying on these stakeholders also takes away from the need to pressure local authorities and the state, and that contributes to their current isolation. Things do not change easily, because it takes education, awareness and working together as a community to make demands:

*The government has a responsibility to create an educated society and informed constituency with health literacy and awareness. But the effort from the government is not sufficient; if the educated people of the community, community representatives and NGOs take initiative and begin working for change then I don’t think it’s a hard thing to achieve*…. *The root of everything is unity. If we are united and stand together to raise these issues, they* [the government] *will have to fulfil our demands.*

## Discussion

The lives of the peoples of the Chittagong Hill Tracts are marked by the isolation and marginalization that comes from being denied an official identity and access to the most basic of social services, ones that are provided for the majority of the dominant ethnic group in the country [36]. The signing of the CHT Accord of 1997 could have been the starting point for change and improvement in the region. However, without the support of international human rights covenants like the UN’s Declaration of Rights of Indigenous Peoples or the ILO’s Indigenous and Tribal People’s Convention of 1989, and without larger national support, little has come of the promises and rights the Accord enshrined. The development of specific policies that aim to reduce disparity and inequality gaps between the dominant ethnic group and that of the people of the CHT will continue to the prorogued because the country does not see it as a priority [37]. The absence of political commitment and structural changes required for real change to occur in order for communities to live better and healthy lives has meant that there is pressure on indigenous communities to create enabling conditions by themselves, without recognising the ongoing injustices which reproduce certain social, economic and political inequalities. International pressure has proven effective in the improvement of health indicators in maternal and child health, and may prove to be a valuable resource in the fight for the rights of the indigenous peoples of Bangladesh.

Participants in our study pointed towards the key role that the Bangladeshi state can and should play in the development of the region. The national and local governments are both duty bearers, and they must live up to their mandate. This means that it is local governments, and not NGOs or international donors that should be providing services and developing pro-equity policies for the population in the Chittagong Hill Tracts [19]. The precarious access to services experienced by the Mro and Tripura show that they are not seen as a priority. Non-governmental organizations have been able to provide some access, but without the large investments that central governments can make, little will change in the long run. However, valuable lessons can be learned from the involvement of NGOs in the region: communities are willing, and even excited about participating, and engaging them might help to strengthen the local social fabric and provide an alternative for development aid. Having communities manage and run water projects can result in the overall development of the village, and can help to identify potential future leaders [38].

For the indigenous people of Bangladesh, precariousness comes not only from being ignored by their state and being denied recognition of their status as a special population group, it also comes from being forced into land that is incapable of sustaining their traditional way of life. Studies show that indigenous people have been able to live off the land and forests in Asia in a sustainable way for centuries [39]. However, the Bengali government has denied the Mro the right to stay in their ancestral lands. The unclear rules and regulations that exist, and the lack of clear property rights make it difficult for these groups to enjoy the use of resources that were once considered theirs. This has also created the conditions that allow the government, the Bangladeshi army and the Bengali settlers to use the local resources for their benefit, and to exclude our study participants from their utilization. As a result, they have little access to safe water, or to forestlands that could provide them with fuel and building materials to build safer and stronger houses. In addition to this scarcity, the participants of our study are forced to share the little access they have with the national army, who has more manpower, better technology and is able to dominate the unarmed locals. This compounds with other social and economic issues, and all contribute to the precariousness and abject poverty in which they live their lives.

Because there is very little access to state or NGO-provided health services in Bandarban, responsibility for health is placed on individuals and their families, who are left to fend for themselves. Attracting healthcare professionals to this area is difficult, because there is little infrastructure or incentives for establishing reliable bases for physicians or nurses [40]. As a result, communities have come to accept any care that is available to them, mostly in the form of traditional healers that live in their vicinity. These healers are not regulated and are not accountable to anyone. Finally, although the communities do have access to health promotion information, and this has contributed to a decline in the prevalence of malaria, typhoid and dysentery, information is not a substitute for curative care.

Mro and Tripura people in Bandarban were promised a system of governance that would reflect their cultural heritage, as well as policies that would allow them to enjoy their rights as citizens of Bangladesh [6, 8, 19, 41]. Almost 20 years after the Accord of 1997, there have been few positive changes in this regard. Citizenship and the rights that come with it are still an abstract promise, and locals are not involved in making decisions about the policies that directly affect them and their lives. What is needed are structural changes, but these require action from the central government [6]. Meanwhile, local level government institutions could fill some of the gaps, and locals perceived them to be the way forward for the overall development of the community. However, participants expressed frustration with these structures. A confusing web of distinctive but overlapping traditional, regional, and national systems of governance with corresponding mechanisms for decision-making further complicate the process for demanding and effecting change.

## Conclusion

Like indigenous people from all over the world, the Tripura and the Mro feel a deep attachment to the land and the nature around them. However, this link is strained by the precarious position they have in regards to their standing within the Bangladeshi state, their access to what should be state-provided services, and to the amount of resources available to them. When this is placed in the social, historical, and political context, we see that the determinants of their marginalization require more than NGOs and international donors. Clear policies that recognize their status as indigenous peoples and that allow for the development of infrastructure and services are needed. Communities have shown that they are aware of their own priorities, and that they value participation. Without the Bangladeshi state’s direct and effective involvement, little will change.
